# Supplementary material for: Clinical value of the fibrosis-4 index in predicting mortality in patients with right ventricular pacing
Source: PLoS One. 2024 Feb 5;19(2):e0294221. doi: 10.1371/journal.pone.0294221 (PMC10843135; doi:10.1371/journal.pone.0294221)
Supplement: Attachment — Submitted filename: Response to Reviewers.docx [file pone.0294221.s002.docx]

January 6, 2024

Emily Chenette

Editor-in-Chief

*PLOS ONE*

Dear Editor:

I wish to re-submit the manuscript titled “**Clinical value of the fibrosis-4 index in predicting mortality in patients with right ventricular pacing.**” The manuscript ID is PONE-D-23-34285.

We thank you and the reviewers for your thoughtful suggestions and insights. The manuscript has benefited from these insightful suggestions. I look forward to working with you and the reviewers to move this manuscript closer to publication in the *PLOS ONE*.

The manuscript has been rechecked and the necessary changes have been made in accordance with the reviewers’ suggestions. The revisions to the text have been highlighted in yellow. The responses to all comments have been prepared and attached herewith.

Thank you for your consideration. I look forward to hearing from you.

Sincerely,

Naoya Inoue

Department of Cardiology, Chutoen General Medical Center

1-1 Shobugaike, Kakegawa, Shizuoka, Japan

Phone No: 090-6581-8948

Fax No: 0537-28-8971

Email Address: [inouenaoya251410@gmail.com](mailto:inouenaoya251410@gmail.com)

Editorial Office Comments:

Please review your reference list to ensure that it is complete and correct. If you have cited papers that have been retracted, please include the rationale for doing so in the manuscript text, or remove these references and replace them with relevant current references. Any changes to the reference list should be mentioned in the rebuttal letter that accompanies your revised manuscript. If you need to cite a retracted article, indicate the article’s retracted status in the References list and also include a citation and full reference for the retraction notice.

Response:

We have checked the reference list and found no retracted papers among the cited articles.

Reviewer #1: The authors revised appropriately. I agree acceptance of this report.

Response:

Thank you very much for taking the time to review our paper amid your busy schedule. We sincerely appreciate your valuable feedback and comments.

Reviewer #2: Dr. Inoue et al. have significantly improved their manuscript based on reviewers’ suggestions. My remaining comments are as follows.

Major:

1. *I appreciate the limitation added, but I am concerned that the study does not sufficiently consider liver disease when using a liver index to predict outcomes in the field of cardiology. I understand that information on liver function is limited, but I believe chart reviews can reveal medical histories at the time of pacemaker implantation, at least with respect to hepatitis B and hepatitis C (from blood tests), and maybe history of liver disease and alcohol use.*

Response:

Thank you very much for your invaluable comments. We also apologize for the lack of information regarding liver diseases. As you pointed out, we have added additional details regarding the results of hepatitis virus testing at the time of pacemaker insertion. Regarding alcohol consumption, we decided to omit this information owing to limited reliability in the medical records, including the frequency, amount, and, in some cases, the presence of alcohol consumption. We sincerely apologize for the inconvenience.

"The number of patients with a positive HCV Ab was significantly different among the three groups; however, post hoc analysis using the Bonferroni method revealed no significant differences between each pair of groups (Tertile 1 vs 2; P = 1, Tertile 1 vs 3; P = 0.091, Tertile 2 vs 3; P = 0.23).”

2. *Table 1: There are 3 groups, so please clarify what the P-values refer to. Is this a 3-group comparison or a comparison between Tertiles 1 and 3? If the former, there is no description of multiple comparisons in the statistical analysis section.*

Response:

Thank you for your comment. We apologize for the lack of clarity in our explanation. We have added the following sentences to the Statistical Analysis section:

"In the comparison of patient characteristics among three groups, we conducted the Kruskal-Wallis test and reported the P-value for overall comparison. To adjust for the significance level in multiple comparisons, we performed post-hoc analyses using Bonferroni correction."

3. *Thank you for adding S2 Table. I believe this is an important table and may be included in the main text, unless there is an upper limit on the number of tables/figures. Core aspects can be included in the Results and Discussion sections of the main text. In particular, 16 of the 38 deaths were due to cancer or infection. Given death within 3 years, some of the 9 patients who died of cancer probably had cancer at the time of pacemaker implantation. This is why baseline information is very important. The authors have very detailed information on CV risk factors in Table 1, so I believe medical charts at the time of pacemaker implantation will reveal any known history of cancer at that time. Cancer is also a CV risk factor.*

Response:

Thank you for your valuable suggestion. We agree with you that the table in S2 Table is important, as you have indicated. Therefore, we have added it as a new Table 3 in the text instead of in the supplemental data. In addition, we have added “Cancer” to the CV risk factors in Table 1.

“Regarding cancer, a cancer related to the liver was not observed as one of the CV risk factors. While a significant overall difference was noted for cancer, Bonferroni analysis showed no significant differences between Tertile 1 vs 2; P = 1, Tertile 1 vs 3; P = 0.62, Tertile 2 vs 3; P = 0.076."

4. *It is reassuring to see that FIB-4 was a significant predictor independent of ALBI. However, the grouping of factors in the multivariable Cox analysis still appears arbitrary to me. I would like to see a multivariable Cox analysis including the following 4 factors:*

*FIB-4, ALBI (or albumin), hemoglobin, and BNP. These were the factors with the lowest P values in Table 1. I would like to know if FIB-4 remains an independent predictor in this analysis.*

Response:

Thank you for your valuable comments. Following your suggestion, we have conducted an additional analysis using the factor with the lowest p-value in Table 1. In this analysis, no statistically significant difference was observed in the hazard ratio between Tertile3 and Tertile1. However, we consider this result to be highly important; therefore, we have added the following text to Table 4:

“Furthermore, the hazard ratio was 2.10 (95% CI: 0.79–5.55, P = 0.13) when adjusted for ALBI score, BNP, and Hb, which were significantly different in the comparison of patient characteristics (Table 4).”

5. *As stated previously, I believe the Discussion should be expanded to discuss various aspects of the study, including possible relationship with non-cardiovascular deaths. The authors have added new analyses as per the reviewers’ suggestions, but have not discussed their new analyses in the Discussion section.*

Response:

Thank you for your comments. We apologize for the insufficient discussion in our part. We have added insights regarding this new analysis in the Discussion section.

“On the other hand, heart failure hospitalization was more frequent in other major adverse cardiovascular events, while deaths related to infections and cancer were more prevalent in Non-CVD. The relationship between the FIB-4 index and heart failure has been reported, specifically suggesting a potential overlap of risk factors or mechanisms between heart failure preserved ejection fraction and liver fibrosis [17]. This association is speculated to remain unaffected even after pacemaker implantation. In Non-CVD, deaths related to liver diseases were not observed, and none of the patients had indications of liver-related cancer at baseline. Nevertheless, in patients with high FIB-4 index, there is a hypothetical possibility of an increased risk of Non-CVD, suggesting the presence of underlying causes leading to liver injury and the systemic impact of inflammatory cytokines due to repetitive injuries [17,18]. However, it is crucial to note that this study is not designed to elucidate these factors, and therefore, these observations remain speculative.”

Minor:

1. *“Ultimately, 201 patients were included in the study.” is stated under Materials and Methods, but this is a result. It is also mentioned at the beginning of the results section, so it can be deleted from Materials and Methods.*

Response:

Thank you for your valuable opinion and apologize for this inaccurate expression. As pointed out, we have removed this text.

2. *EZR software: provide the version number.*

Response:

Thank you for pointing this out to us. We have added it.

3. *Table 1: “ECG after RVP implant” should be “ECG after RVP implantation”*

Response:

Thank you for your comments. We have corrected the word that you pointed out.

4. *Table 1: “Atrium” should be “Atrial” to be consistent with “Ventricular” which comes up later on.*

Response:

Thank you for your comments. We have corrected the word that you pointed out.

5. *S2 Table: Provide a footnote with abbreviations. Also, it would be easier to interpret if the causes of death were ordered by frequency.*

Response:

Thank you for your valuable advice. As mentioned earlier, we inserted the S2 table in Table 3 into the main text and arranged the causes of death in order of frequency. We have also added annotations for these abbreviations.
