# Supplementary material for: Clinical value of the fibrosis-4 index in predicting mortality in patients with right ventricular pacing
Source: PLoS One. 2024 Feb 5;19(2):e0294221. doi: 10.1371/journal.pone.0294221 (PMC10843135; doi:10.1371/journal.pone.0294221)
Supplement: Attachment — Submitted filename: Response to Reviewers.docx [file pone.0294221.s003.docx]

January 21, 2024

Emily Chenette

Editor-in-Chief

*PLOS ONE*

Dear Editor:

I wish to re-submit the manuscript titled “**Clinical value of the fibrosis-4 index in predicting mortality in patients with right ventricular pacing.**” The manuscript ID is PONE-D-23-34285.

We thank you and the reviewers for your thoughtful suggestions and insights. The manuscript has benefited from these insightful suggestions. I look forward to working with you and the reviewers to move this manuscript closer to publication in the *PLOS ONE*.

The manuscript has been rechecked and the necessary changes have been made in accordance with the reviewers’ suggestions. The revisions to the text have been highlighted in yellow. The responses to all comments have been prepared and attached herewith.

Thank you for your consideration. I look forward to hearing from you.

Sincerely,

Naoya Inoue

Department of Cardiology, Chutoen General Medical Center

1-1 Shobugaike, Kakegawa, Shizuoka, Japan

Phone No: 090-6581-8948

Fax No: 0537-28-8971

Email Address: [inouenaoya251410@gmail.com](mailto:inouenaoya251410@gmail.com)

Editorial Office Comments:

I appreciate the effort and time that the authors spent on revisions of the paper. However, I agree with Reviewer#2 that the authors should be more cautious on the interpretatio when the ajusted results show no associations between FIB-4 and the endpoint after adjustment of ALBI, BNP, and hemoglobin, which were different among groups at baseline.

Would you please revise the interpretation of the results? For example, "However, the results of our study revealed that the FIB-4 index was independently associated with all-cause mortality" was a misinterpretation in this case. Please also discuss about the possible explaination why the association was neutralized by those adjustment.

Response:

Thank you for your important comments. We agree with the significance of the observations made by Reviewer #2. Subsequently, we have attempted to address the identified issues and prepared a comprehensive response to Reviewer #2, approaching it from various angles and to the best of our ability. Thank you for your comment.

Reviewer #2: The authors have again significantly revised their manuscript. While I appreciate their hard work, the revisions have led to significant concerns on my part.

Major:

1. *1. The authors found that FIB-4 was NOT an independent predictor of shorter OS when controlling for ALBI, BNP, and hemoglobin. The authors do not show which of these 3 factors were independent predictor of shorter OS, but this at least means that a model with albumin, bilirubin, BNP, and hemoglobin is a better predictor than that with AST, ALT, age, and platelets (the FIB-4 index). This is not discussed at all, but this result should not be taken lightly, as it may significantly undermine the value and conclusions of this whole study.*

*2. There are many instances where the authors emphasize findings that support their hypothesis and downplay findings that do not support their hypothesis. I am concerned that the manuscript may mislead the reader to believe that the FIB-4 index is more valuable than it really is in the context of patients undergoing right ventricular pacing.*

*3. As a result of the added analysis, the clinical implications of this study and the FIB-4 index in the studied cohort have also become unclear.*

Response:

Thank you very much for your exceptionally important comments. First, we sincerely appreciate the accuracy of your observations and apologize for the part of our conclusion that was phrased in a way that could lead to misunderstanding. Accordingly, we have revised and removed the relevant sections.

As an initial consideration in response to the above points, we began by detailing the results of Cox analyses for various combinations, including ALBI score, BNP, Hb, and others.

| **All-cause mortality** | **Hazard ratio**  **(95% CI)** | ***P*-value** |
| --- | --- | --- |
| **Unadjusted**  **Tertile 1 (FIB-4 index ≤ 2.17)**  **Tertile 2 (2.18 ≤ FIB-4 index ≤ 3.28)**  **Tertile 3 (FIB-4 index ≥ 3.29)** | Reference  0.81 (0.25-2.57)  4.75 (2.05-11.0) | Reference  0.73  <0.001 |
| **Model 1: Adjusted for ALBI score**  **Tertile 1 (FIB-4 index** ≤ **2.17)**  **Tertile 2 (2.18 ≤ FIB-4 index ≤ 3.28)**  **Tertile 3 (FIB-4 index** ≥ **3.29)**  **ALBI score** | Reference  0.67 (0.21-2.13)  3.25 (1.33-7.91)  2.87 (1.33-6.19) | Reference  0.49  0.009  0.007 |
| **Model 2: Adjusted for BNP**  **Tertile 1 (FIB-4 index**≤ **2.17)**  **Tertile 2 (2.18 ≤ FIB-4 index ≤ 3.28)**  **Tertile 3 (FIB-4 index** ≥ **3.29)**  **BNP** | Reference  0.63 (0.19-2.09)  3.35 (1.36-8.24)  1.00 (1.00-1.00) | Reference  0.45  0.008  0.020 |
| **Model 3: Adjusted for Hb**  **Tertile 1 (FIB-4 index**≤**2.17)**  **Tertile 2 (2.18 ≤ FIB-4 index ≤ 3.28)**  **Tertile 3 (FIB-4 index** ≥ **3.29)**  **Hb** | Reference  0.67 (0.21-2.14)  3.18 (1.29-7.84)  0.78 (0.63-0.96) | Reference  0.50  0.011  0.020 |
| **Model 4: Adjusted for ALBI score and BNP**  **Tertile 1 (FIB-4 index**≤ **2.17)**  **Tertile 2 (2.18 ≤ FIB-4 index ≤ 3.28)**  **Tertile 3 (FIB-4 index** ≥ **3.29)**  **ALBI score**  **BNP** | Reference  0.55 (0.16-1.81)  2.54 (1.00-6.45)  3.27 (1.22-8.75)  1.00 (0.99-1.00) | Reference  0.32  0.048  0.018  0.094 |
| **Model 5: Adjusted for ALBI score and Hb**  **Tertile 1 (FIB-4 index**≤ **2.17)**  **Tertile 2 (2.18 ≤ FIB-4 index ≤ 3.28)**  **Tertile 3 (FIB-4 index** ≥ **3.29)**  **ALBI score**  **Hb** | Reference  0.61 (0.19-1.95)  2.64 (1.04-6.71)  2.28 (1.00-5.20)  0.84 (0.67-1.05) | Reference  0.40  0.040  0.048  0.14 |
| **Model 6: Adjusted for BNP and Hb**  **Tertile 1 (FIB-4 index**≤ **2.17)**  **Tertile 2 (2.18 ≤ FIB-4 index ≤ 3.28)**  **Tertile 3 (FIB-4 index** ≥ **3.29)**  **BNP**  **Hb** | Reference  0.56 (0.16-1.85)  2.55 (0.99-6.55)  1.00 (0.99-1.00)  0.81 (0.65-1.01) | Reference  0.34  0.051  0.074  0.070 |
| **Model 7: Adjusted for ALBI, BNP, and Hb**  **Tertile 1 (FIB-4 index**≤ **2.17)**  **Tertile 2 (2.18 ≤ FIB-4 index ≤ 3.28)**  **Tertile 3 (FIB-4 index** ≥ **3.29)**  **ALBI score**  **BNP**  **Hb** | Reference  0.50 (0.15-1.67)  2.10(0.79-5.55)  2.82 (1.03-7.74)  1.00 (0.99-1.00)  0.85 (0.68-1.07) | Reference  0.26  0.13  0.042  0.12  0.18 |

Based on the above results, we also agree with your observation that there were influences from ALBI score, BNP, and Hb. Subsequently, in response to the points raised in Question 1, we conducted ROC analysis to compare the AUC and assess whether the models for ALBI score, BNP, and Hb are superior predictive factors for the FIB-4 index.

PropensityScore.GLM.1: ALBI+BNP+Hb model

As a result, the AUC and 95% confidence intervals for each were as follows: FIB-4 index: 0.727 (0.62-0.82) and ALBI+BNP+Hb model: 0.718 (0.61-0.81). The P-value for the AUC comparison was 0.86, indicating no significant superiority of the ALBI+BNP+Hb model as a predictive factor. However, we also observed that the FIB-4 index did not demonstrate superiority as a predictive factor, and we agree with your assessment.

Subsequently, in accordance with your opinion, we re-evaluated the clinical value of the FIB-4 index in the group undergoing right ventricular pacing. Upon considering the Cox model, when controlled for ALBI and Hb (Model 5), significant differences in FIB-4 remained. However, with the introduction of BNP in Model 6, the significance of the FIB-4 index was no longer observed.

Therefore, to investigate the differences when using the BNP and FIB-4 index as predictive factors, we conducted a comparative assessment using Kaplan-Meier analysis. We will present the results of the Kaplan-Meier analysis, stratifying BNP into tertiles, similar to the FIB-4 index.

The results of the log-rank test were P<0.001 for the FIB-4 index and P=0.008 for BNP, both of which indicated significant differences. However, focusing on the proportional hazards assumption from this graph, it appeared that the hazard changed over time for the proportional hazards test (FIB-4 index, P=0.09; BNP, P=0.035). Therefore, considering concerns about proportional hazards during this period, we conducted a reassessment specifically for a duration of 1000 days (approximately three years).

As a result, the P-values were 0.0014 for the FIB-4 index and 0.16 for BNP, indicating that over approximately 3 years, only the FIB-4 index showed statistical significance. In the Cox proportional hazards analysis, comparing Tetile3 to Tetile1 as the reference, the hazard ratio for the FIB-4 index was HR=3.68, P=0.010, and for BNP, HR=2.87, P=0.070. The proportional hazards assumption was also confirmed, and neither the FIB-4 index nor BNP showed rejection of proportional hazards (P=0.33).

Based on a comparison with BNP, the FIB-4 index was considered to have clinical value as a predictor of mortality, potentially being able to predict earlier mortality events.

In your first comment (Reviewer #2), you suggested the following:

" The authors are proposing a cut-off FIB-4 index of 3.75 in their ROC curve. Therefore, they should present data in 2 groups (FIB-4 index >= 3.75 and < 3.75), not on the top and bottom tertiles. (Also, there is no significant difference between the first and second tertiles, perhaps suggesting that they can be combined.)"

Therefore, we conducted a similar Cox analysis between the two groups using this cut-off value.

| **All-cause mortality** | **Hazard ratio**  **(95% CI)** | ***P*-value** |
| --- | --- | --- |
| **Unadjusted**  **FIB-4 index** ≥ **3.75** | 7.46 (3.82-14.5) | <0.001 |
| **Model 1:Adjusted for ALBI**  **FIB-4 index** ≥ **3.75**  **ALBI score** | 6.34 (3.23-12.5)  3.78 (1.64-8.71) | <0.001  0.0017 |
| **Model 2: Adjusted for BNP**  **FIB-4 index** ≥ **3.75**  **BNP** | 5.83 (2.91-11.6)  1.00 (1.00-1.00) | <0.001  0.031 |
| **Model 3: Adjusted for Hb**  **FIB-4 index** ≥ **3.75**  **Hb** | 6.25 (3.16-12.3)  0.75 (0.61-0.91) | <0.001  0.004 |
| **Model 4: Adjusted for ALBI score and BNP**  **FIB-4 index** ≥ **3.75**  **ALBI score**  **BNP** | 5.17 (2.56-10.4)  3.42 (1.26-9.23)  1.00 (0.99-1.00) | <0.001  0.015  0.14 |
| **Model 5: Adjusted for ALBI score and Hb**  **FIB-4 index** ≥ **3.75**  **ALBI score**  **Hb** | 5.89 (2.97-11.6)  2.76 (1.12-6.78)  0.82 (0.66-1.02) | <0.001  0.26  0.078 |
| **Model 6: Adjusted for BNP and Hb**  **FIB-4 index** ≥ **3.75**  **BNP**  **Hb** | 5.24 (2.59-10.6)  1.00 (0.99-1.00)  0.79 (0.64-0.97) | <0.001  0.20  0.030 |
| **Model 7: Adjusted for ALBI, BNP, and Hb**  **FIB-4 index** ≥ **3.75**  **ALBI score**  **BNP**  **Hb** | 4.85 (2.38-9.88)  2.74 (0.97-7.66)  1.00 (0.99-1.00)  0.84 (0.67-1.05) | <0.001  0.054  0.25  0.12 |

In this analysis, the predictive value of the FIB-4 index remained consistent even after adjusting for ALBI score, BNP, and Hb. Based on these findings, we have revised our conclusions as follows:

' In patients with pacemakers, the FIB-4 index may have value as a predictor of earlier all-cause mortality, with a cutoff value of 3.75.'

We attribute the clinical significance to this study and the FIB-4 index.

Your invaluable insights prompted us to reconsider the clinical significance of this research, leading to the revision of our conclusions. We sincerely appreciate your invaluable input and have incorporated it into our responses.
